# Fifth International Biannual Evolution and Ecology of Cancer Conference (Cooperation, Conflict and Parasitism) meeting report—Wellcome Genome Campus, Hinxton, UK

**DOI:** 10.1111/eva.12862

**Published:** 2019-11-01

**Authors:** Nynke Raven, Georgina Bramwell, Rodrigo Hamede, Frédéric Thomas, Beata Ujvari

**Affiliations:** ^1^ School of Life and Environmental Sciences Deakin University Geelong Vic Australia; ^2^ School of Natural Sciences University of Tasmania Hobart TAS Australia; ^3^ CREEC UMR IRD 224‐CNRS 5290‐Université de Montpellier Montpellier France

**Keywords:** cancer, conflict, cooperation, ecology, evolution, parasitism

## Abstract

The fifth biannual conference of the International Society of Evolution and Ecology of Cancer (ISEEC) was held between the 17th and 19th of July 2019 in Hinxton (UK) at the Wellcome Genome Campus. The main theme of the conference: cooperation, conflict and parasitism reflected our growing understanding of the role cancer has played in the evolution of multicellular organisms, as well as the urgent need of translating these Darwinian processes to treatment strategies. Below we provide a brief summary of each plenary sessions and other oral presentations, to bring the conference to the broader audience of evolutionary biology and applications.

## 1 INTRODUCTION

The fifth biannual conference of the International Society of Evolution and Ecology of Cancer (ISEEC) was held between the 17th and 19th of July 2019 in Hinxton (UK) at the Wellcome Genome Campus. The main theme of the conference: cooperation, conflict and parasitism reflected our growing understanding of the role cancer has played in the evolution of multicellular organisms, as well as the urgent need of translating these Darwinian processes to treatment strategies.

Cancer, the uncontrolled proliferation of cells, is an ancestral disease that presented a major evolutionary hurdle during the transitioning from unicellular to metazoan life. Cells that were able to give up their own reproductive interests, cooperate, control and suppress the proliferation of selfish cells, opened up the possibility of multicellular organism to appear, to evolve and to thrive.

Cancer, the disease as we know it now, occurs when, during the host’s lifetime certain cells lose their cooperative behaviour, acquire traits of unlimited proliferation and the propensity to invade, and thus become malignant. Selfish traits emerge via natural selection acting at multiple levels (cheaters gaining individual advantage over the cooperative group that they exploit), and thus, Darwinian selection drives cancer cells to escape immune attack, metastasize, resist therapies and sometimes even to become contagious.

The aim of the biannual meeting therefore was to bring together clinicians, theoreticians and evolutionary biologists from different disciplines across the world (>150 attendants from >20 countries) to present the latest research developments in the field. The conference organizers placed special emphasis on and achieved gender and career stage balance amongst the invited speakers.

The conference discussed the evolutionary and ecological aspects of cell competition, cooperation, conflict and parasitism in tumour development and progression as well as the impact of cancer on organismal form and function. The last day of the conference was dedicated to the emerging and exciting field of transmissible cancers. During the conference, special focus was placed on how Darwinian approaches can be applied to cancer treatment via mathematical modelling, experimental designs and by harnessing knowledge from billions of years of evolution since multicellular organisms have emerged.

Below we provide a brief summary of each plenary sessions and other oral presentations, to bring the conference to the broader audience of evolutionary biology and applications.

## 2 SUMMARY OF PRESENTATIONS

The fifth biannual conference of the International Society of Evolution and Ecology of Cancer was opened on the 17th of  July 2019 by Dr Kenneth J. Pienta MD (John Hopkins University, USA) with a keynote talk entitled ‘How understanding evolution and ecology can change our patient care and lead to new scientific insights’. Dr Pienta explained the dynamics of how cancer cells grow and spread throughout the body, before sharing a new exciting discovery of polyploid multinucleated giant cells, which may be the key to how cancer survives and evolves resistance against current/traditional therapeutic treatments. The discovery of the giant cells may give researchers a focus to better understand how to prevent therapeutic resistance in cancer evolution.

Session one—cooperation and conflict in multicellularity. Chaired by Dr Carlo Maley (Arizona State University, USA).

Dr Michelle M. Leger’s (Institute of Evolutionary Biology, Spain) presentation was titled ‘What protists can tell us about multicellularity, cell death, and the origins of animals’. Dr Leger’s research used unicellular organisms (holozoan protists) to gain insight into the origins of various traits and features of closely related multicellular animals. For example, programmed cell death is an ancestral trait found in single cell protists, but apoptosis is a specific trait of multicellular organisms.

Dr Ferran Muinos (Institute of Research in Biomedicine, Barcelona) discussed how ‘Systematic learning from tumour genomes enables the identification of cancer genes and driver mutations’. Dr Muinos is interested in understanding which somatic cellular mutations drive tumour development. Using bulk sequencing of single nucleotide variants, his aim is to develop new computational methods that understand mutations in tumours allow for the detection of positive selection. His talk demonstrated that tens of thousands of primary and metastatic cancers have been analysed, using multiple methods, to train the sophisticated computer analysis programme, which has improved in accuracy and allowed the ability to detect mutations providing selective advantage to tumours.

Dr Aurora M. Nedelcu (University of New Brunswick, Biology Department, Canada) presented: ‘Cooperation is costly, but so is cheating: Implications for the evolution of multicellularity and cancer’. Dr Nedelcu discussed advantages and disadvantages of altruistic behaviour, and cheating in somatic cells, focusing on volvocine algae and tumour suppressor genes regA and RLS1, showing that the expression of these genes conferred advantages, including higher replication, in normal environments. However, these cheater cells are more sensitive to environmental change and are at a disadvantage compared to altruistic somatic cells, in more extreme environments.

The first session was concluded by Dr Lisa M. Abegglen from the Huntsman Cancer Institute (USA) with the title: ‘Enhanced DNA damage response in species with low cancer prevalence rates’. Dr Abegglen discussed the use of primary fibroblasts to test the efficiency of tumour responses in animals. The research showed strong apoptotic responses to cancer in animals with low cancer prevalence, but weak or no apoptotic responses in animals with naturally high tumour/cancer prevalence. Dr Abegglen proposed that enhanced DNA damage response could be an important mechanism in cancer suppression.

Session two—cellular competition. Chaired by Dr Athena Aktipis (Arizona State University, USA).

The first speaker, Dr Ginés Morata (Centre for Molecular Biology, Spain) presented his research on ‘Cell competition: a mechanism of cell selection in animal tissues’. Dr Morata discussed cell competition and how the loser, while viable, undergoes apoptosis by JNK pathway. Cells carrying oncogenic mutations are often detected as ‘the loser’ displaying a link between cell competition and tumorigenesis.

Dr Kairbaan Hodivala‐Dilke (Barts Cancer Institute, UK) work was on ‘Cancer associated fibroblast‐FAK regulates malignant cell metabolism’. Dr Hodivala‐Dilke discussed how cancer progresses and grows, not only by cancer type, but via the tumour and host microenvironment. One factor contributing to this growth is the stromal focal adhesion kinase expression (FAK), which at low levels can allow an increase in tumour growth and aerobic glycolysis. Increased levels of FAK in human trials translated to better survival of cancer patients.

Dr Eszter Lakatos (Queen Mary University of London, UK) presentation was titled: ‘The evolutionary processes shaping the neoantigen landscape during tumour growth’. Dr Lakatos, a self‐confessed data parasite, is interested in understanding how tumours can evolve by mutations and how to measure negative selection within tumours. Using mathematical models, negative selection can be detected; however, strong negative selection can decrease mutations to an extent that it appears as neutral selection to the models.

Dr James DeGregori (Department of Biochemistry and Molecular Genetics, University of Colorado Anschutz Medical Campus, USA) discussed ‘An evolutionary understanding for how aging determines cancer incidence’. Dr DeGregori’s studies have uncovered molecular mechanisms underlying oncogene‐driven adaptation to aged tissue environments and found that anti‐inflammatories within an aged population reduced the growth of cancer. These studies indicate the need for age‐dependent selection and life history‐dependent evolution to be incorporated into cancer incidence modelling, and for cancer treatment strategies that involve altering tissue microenvironments.

Following the second session, the first poster lightening talks took place, followed by a poster session.

The second day began with session three—Math models of cooperation and competition in cancer. Chaired by Dr Trevor Graham (Barts Cancer Institute, UK)

The first speaker, Dr Diana Fusco (University of Cambridge, UK) presented the topic of ‘What can microbial colonies teach us about spatial intra‐tumour heterogeneity’. Dr Fusco modelled and discussed how space and mutations effect tumours and how the cancer cells within the tumours respond to their crowded environment. This response can alter both proliferation rates and genetic drift, removing diversity. The probability of the rise of resistance during tumour incubation was also modelled, allowing insight into evolution occurring within a population.

Dr Katerina Stankova’s (Maastricht University, the Netherlands) talk focused on ‘Cancer therapy: changing the game’. Dr Stankova explored cancer in the framework of game theory, using the knowledge of how cancer responds to modern therapies to formulate more effective and strategic treatments. Rather than using the maximum tolerable dose of treatment, which stimulates resistance in the tumour causing it to evolve following Darwinian dynamics and to grow back more aggressively, to use the minimum effective dose to control the environment around the tumour, allowing the natural cellular heterogeneity within the tumour to control and restrict therapeutic resistance. This strategy allows the physician to control and dictate the direction of the game.

Máté Kiss, PhD candidate (Eötvös Loránd University, Hungary), presented a very interesting topic on ‘Tissue hierarchy in plants can efficiently minimize somatic evolution and act as a functional germline’. Kiss discussed how plant growth is governed by cell divisions in the apical meristem, and how plants are able to limit the accumulation of somatic mutations, even in areas of high cell division. Plants can slow the stem cell divisions in the apical meristem and exponentially increase divisional rate of cells. The results show tissue hierarchies underlying plant growth can minimize the accumulation of somatic mutations.

Dr Wolfram Möbius (University of Exeter, UK) talk was titled: ‘Two layers of chance associated with spatially expanding populations: How environmental heterogeneity and demographic noise shape genetic diversity’. Dr Moebius discussed the importance of spatial structure and local environment on the evolutionary dynamics of growing and expanding cell populations. This spatial environment can stimulate cell adaptation and impact diversity of cellular lineages in cell communities and can, in some situations, have a higher impact than intracellular competition. The modelling work of Dr Moebius and colleagues provides a framework to rationalize and characterize the effects of ‘geometry‐enhanced genetic drift’ on large cell populations.

Session four—cooperation and cancer. Chaired by Dr Elizabeth Murchison (University of Cambridge, UK).

The first speaker for this session, Dr Michalina Janiszewska (The Scripps Research Institute, USA), showed that ‘Breast cancer metastasis is driven by clonal cooperation and immune microenvironment interactions’. Dr Janiszewska educated us about her experimental breast cancer model which allowed two well‐known driver clones to promote growth of a primary tumour and following metastasis. They identified neutrophils as key players in formation and communication within metastasis, and although neutrophil depletion decreased subsequent lung metastasis, it showed little effect on the primary tumour. Dr Janiszewska’s research is shedding light on how changes in local and systematic immune environment could be useful in predicting cancer growth and could potentially be exploited to prevent metastasis.

Dr Alexander Anderson (Moffitt Cancer Center, USA) talked about ‘Personalized adaptive therapies for metastatic melanoma’. Dr Anderson discussed the background and benefits of adaptive cancer therapy, a new type of therapy that uses lower doses of drugs more strategically. Adaptive treatment accounts for evolution of cancer resistance during drug treatment. Cancer resistance is only beneficial in the presence of treatment, without the treatment, sensitive, vulnerable cells have intracellular competitive advantage and will assist in controlling the spread of harmful, resistant cells. The higher effectiveness of adaptive therapy shows that evolution should not be dismissed in cancer treatment.

Dr Alex Cagan (Wellcome Sanger Institute, UK) presentation focused on ‘Comparison of somatic mutational processes across mammalian species’. Variation in somatic mutation rate across the animal kingdom may give insights into Peto’s paradox. Long‐lived animals show lower rates of somatic mutation, which could indicate more investment in effective cancer preventative mechanisms, and consequently could contribute to a slower rate of ageing. Dr Cagan’s research combining life‐history records and sequencing data provides insights into mechanisms underlying somatic mutation and how they contribute to both cancer and ageing.

Dr Khalid Abdul‐Jabbar (Institute of Cancer Research, UK) presented: ‘Deep learning the immune microenvironment complexity shaping lung cancer evolution’. Dr Abdul‐Jabbar examined the spatial configuration of the immune microenvironment and tissue architectural heterogeneity within tumours and successfully used deep learning to predict cancer evolution. Showing that spatial variability and context of immune presence in the tumour microenvironment is important in evolutionary selection and therefore should be acknowledged when considering clinical cancer phenotypes.

Dr Sarah Amend’s (John Hopkins University, USA) talk was titled: ‘Cancer cell optimal foraging: motile foragers are the actuators of metastases’. Optimal foraging theory gives insights into the conditions within the primary tumour that could stimulate metastases. While cells leaving the primary tumour to forage (moving) face high risk, high reward, the movement itself selects for cancer cells with high metastatic potential. Understanding and moderating modern cancer treatments that stimulate optimal foraging conditions involving cell movement could help decrease cancer metastases and resistance in the future.

Session four closed with a keynote lecture by Dr Mel Greaves (Institute of Cancer Research, UK) presenting on ‘Cancer transmission in humans’. Dr Greaves discussed unique cases of transmission of cancer between humans, suggesting cancer should be classified as a parasite. Rare cases involve accidents including organ donors, surgeons, and transmission between mother and foetus, and the majority of these rare cases are underlined by some type of natural or artificial immune suppression. He also noted an interesting addition of transfusion between twins sharing a placenta.

Session five—evolution and adaptability. Co‐chaired by Dr Carlo Maley and Dr Athena Aktipis

Dr Michael Lynch (Arizona State University, USA) talked about the ‘Evolution of intracellular error rates and the origins of cancer’. The drift‐barrier hypothesis suggests that the limits of natural selection are dictated by the power of random genetic drift, rather than molecular or cellular constraints. The population‐genetic environment constrains evolutionary paths in different phylogenetic lineages and helps define possible adaptation patterns seen at a cellular level. Both multicellularity and population size can affect the strength of this drift, in some cases allowing for fixation or selective sweeps.

Dr Jacob G. Scott (Cleveland Clinic, USA) presented a talk on ‘Speeding up evolution: exploiting contingencies on clinically relevant timescales’. Dr Scott discussed the theory of treating cancer by using collateral sensitivity, i.e. steering the evolution of cancer cells to become susceptible to treatment by applying different drug combinations. Collateral sensitivity treatment requires understanding the adaptation each drug stimulates from cancer, how long this adaptation lasts in the absence of treatment and which drugs the cancer becomes susceptible to as a result of treatment. Scientists can use the knowledge to judge time lags between administering drugs and drug switching, controlling the adaptation, to keep the cancer susceptible and reasonably controlled.

Dr William Cross (Barts Cancer Institute, UK) discussed ‘Clonal evolution and stabilizing selection in colon adenomas and carcinomas’. Cancer karyotype, with its driver mutation complement, while overlooked in research, is a strong driver of selection in cancer. Selection occurring during carcinogenesis is complex and for optimal cancer fitness, uses stabilizing selection to constrain a number of cancer karyotypes.

Dr Luiza Moore (Wellcome Sanger Institute, UK) talked about the ‘Mutational landscape of normal endometrial epithelium’. Endometrial epithelial glands are mostly clonal populations of cells with higher mutational burden when compared with normal cells, although lower than endometrial cancers. The mutational processes occurring in this tissue is relatively homogenous with only occasional outliers. Age has a positive association with the accumulation of driver mutations while parity has a negative association, and the distinct mutational landscape suggests the presence of early positive selection in endometrial tissue.

Dr Robert J. Downey’s (Memorial Sloan Kettering, UK) talk was on ‘Quantification of intra‐tumour spatial heterogeneity in lung adenomas’. Using a combination of multi‐region, high‐throughput mass spectrometry and computational analysis, distinct spatial patterns of intra‐tumour heterogeneity were determined using the extent of richness and evenness of molecular features and their correlation with geographic locations. The findings provide novel insights into the clinical impact of geographic diversification in lung adenocarcinoma. The methods used demonstrate the extent of intra‐tumour heterogeneity and diversification and their use as a novel clinical predictor of survival, that could be applied to any tumour type.

Following session five, the second lightening talks took place which was finished by a poster session.

The third and final day began with a keynote lecture by Dr Athena Aktipis on transmissible cancers and the evolution of cellular cheating: How multicellularity evolved to protect cellular cooperation and limit invasion.

Session six—Transmissible cancer. Co‐chaired by Dr Trevor Graham and Dr Elizabeth Murchison.

The session began with Dr Michael J. Metzger (Pacific Northwest Research Institute, USA) who presented a talk on ‘Contagious cancer in clams! Genetics and evolution of bivalve transmissible neoplasia’s and their hosts’. Transmissible cancers within bivalves are a relatively newly discovered occurrence. By investigating genomic changes in the evolution of the transmissible neoplastic lineage within two populations, changes within the early stages of the lineage and those after divergence can be observed. A retrotransposon (*Steamer*) was found to be expressed and amplified within the cancer linage.

Dr Beata Ujvari’s (Deakin University, Australia) talk focused on ‘Somatic cell parasitism: contagious cancer cell lines as ultimate parasites’. By observing cancer cells as parasites, it is possible to predict how cancer cell transmission occurs and identify species with the higher chance of transmissible cancers to occur in. Cancer cells and parasites share phenotypic traits and adaptations (such as structural changes to facilitate mobility and genomic modifications) that are most likely the result of convergent evolution. Some species has the characteristics and phenotypes (e.g. the ability of bivalve mitochondria to switch to anaerobic metabolism) that provides the ideal environment for (transmissible) malignant cells to emerge and to thrive in.

Adrian Baez‐Ortega (University of Cambridge, UK) presented ‘The evolutionary history of a transmissible cancer’. By utilizing phylogenetic analysis, it is possible to view the history and spread of canine transmissible general tumour disease (CTVT) while analysing the diversity and evolution around the world. The potential use of long‐lived clonal organisms acting as biomarkers was found, along with the evidence supporting neutral generic drift of the tumour.

Alicia L. Bruzos (University de Santiago de Compostela, Spain) talked about ‘Scuba cancers: finding the genetic causes of contagious metastases under the sea’. The Scuba Cancers Project aims to identify possible genetic causes of transmissible haemic neoplasia within cockles of the Atlantic coast of Europe. Whole mitochondrial genome sequencing supported the existence of five clonal lineages of the neoplasia, possibly more.

Dr Rodrigo Hamede (University of Tasmania, Australia) discussed ‘Cancer, behaviour, sex and transmission: infection status and tumour load affect contact rates, mating interactions and social network structure in wild Tasmanian devils’. Sickness behaviour, while documented in connection with various diseases, has been difficult to study in wild animals with cancer. Tasmanian devils show changes in inter‐individual interaction when infected with DFTD, suggesting DFTD exerts selective pressures on behaviours associated with infection risk, and could be a force driving adaptive processes for the long‐term coexistence of devils and DFTD tumours.

Dr Elizabeth Murchison (University of Cambridge, UK) focused on the ‘Origins and evolution of transmissible cancers’. Using genetical analysis, the origins and evolution of the long‐lived cancer lineages of DFTD and CTVT can be observed. When focusing on CTVT, it is known to be sexually transmitted and is easily treated with chemotherapy as it does not metastasize. Dr Murchison discussed the frequency of mitochondrial horizontal transfer within CTVT.

Dr Martin Santamarina Garcia’s (University de Santiago de Compostela, Spain) talk was about ‘The evolutionary dynamics of somatic retrotransposition in a millennial cancer lineage’. Preliminary results were shared on the evolutionary dynamics of somatic retrotransposition in CTVT. Using a combination of well‐established bioinformatic algorithms, phylogenetic analyses and long‐read sequencing for verification, he found evidence to support what genetical conditions could be required for a transmissible cancer lineage to survive an extended time.

The final speaker of the conference was Maurine Hammel (Montpellier University, France) who concluded the conference with the talk ‘Genotype typing of transmissible cancer in the *Mytilus edulis* complex of species’. Bivalve transmissible cancer originating from *Mytilus trossulus* was located within the *M. edulis* of Europe. Single nucleotide polymorphism genotyping was used to locate genetic chimerism and locate any polymorphism amongst the neoplastic samples suggesting that the cancer emerged from a hybrid or ancestral population.

The conference concluded with general closing remarks and awards for the best posters shown at the conference. The award for best poster was awarded to Dr Ryan O Schenck (University of Oxford and Moffitt Cancer Centre) for his poster entitled ‘How homeostasis limits Keratinocyte evolution’. The fifth International Conference of the Evolution and Ecology of Cancer was the first to take place outside of the United States. This shows how much the field of scientific research in relation to cancer within both medical and ecological environments is growing with exciting progress.

## CONFLICT OF INTEREST

None declared.

